# Short-term memory trace mediated by termination kinetics of olfactory receptor

**DOI:** 10.1038/srep19863

**Published:** 2016-02-01

**Authors:** Sean Michael Boyle, Shane McInally, Sana Tharadra, Anandasankar Ray

**Affiliations:** 1 Genetics, Genomics and Bioinformatics Program, University of California, Riverside, California, CA 92521; 2 Department of Entomology, University of California, Riverside, California, CA 92521; 3 Center for Disease Vector Research, University of California, Riverside, California, CA 92521; 4 Institute of Integrative Genome Biology, University of California, Riverside, California, CA 92521.

## Abstract

Odorants activate receptors in the peripheral olfactory neurons, which sends information to higher brain centers where behavioral valence is determined. Movement and airflow continuously change what odor plumes an animal encounters and little is known about the effect one plume has on the detection of another. Using the simple *Drosophila melanogaster* larval model to study this relationship we identify an unexpected phenomenon: response to an attractant can be selectively blocked by previous exposure to some odorants that activates the same receptor. At a mechanistic level, we find that exposure to this type of odorant causes prolonged tonic responses from a receptor (Or42b), which can block subsequent detection of a strong activator of that same receptor. We identify naturally occurring odorants with prolonged tonic responses for other odorant receptors (Ors) as well, suggesting that termination-kinetics is a factor for olfactory coding mechanisms. This mechanism has implications for odor-coding in any system and for designing applications to modify odor-driven behaviors.

Animals are constantly exposed to a changing landscape of complex olfactory cues and decision-making within such complex odor environments is critical for navigation behaviors such as finding food, determining oviposition sites, avoiding predators, and identifying mates. Behavioral outcome is thought to depend not only on detection of odor cues by the olfactory system but also prior learning experiences. Little is understood about detection, learning and behavior in rapidly changing odor environments. In most animals, the olfactory system is responsible for detecting chemical cues in the environment and conveying that information to the brain so that valence – attractiveness or repellency - can be determined. In both vertebrates and flies, primary olfactory neurons (ORNs) are highly specialized cells that typically express single or few receptor proteins^1^,^2^,^3^. ORNs expressing the same receptor project their axons to the same glomeruli in the brain. In *Drosophila*, the receptor to glomerulus map is highly stereotypical and has been extensively studied. A given odorant may activate several receptor types and therefore several ORN classes to produce a distinct pattern of activation across the glomeruli of the antennal lobe^4^. This activation pattern presumably leads to a valence decision and behavioral output.

In order to further examine how individual receptors contribute to an animal’s behavioral response to changing odor concentrations, we have employed the simple larval olfactory model. *Drosophila* larvae only have 21 ORNs on each side of the head with their dendrites housed in the dome sensillum. Each of these 21 sensory neurons is thought to belong to a different class and express a different receptor^2^,^5^,^6^. Typically, a “tuning” Or is singularly expressed along with the obligate co-receptor Orco^7^. As in adults, different ORNs project their axons to different glomeruli in the larval antennal lobe (LAL). The use of optogenetic techniques has demonstrated that activation of a single ORN type can effectively drive chemotaxis behavior^8^. Most larval ORNs direct attractive behavior, while only a few mediate repellency^6^,^8^,^9^. However, the associated valences of all larval ORNs and how activation of multiple channels impacts behavior have yet to be determined.

The Or42b expressing neuron confers one of the strongest attractions of the 21 receptor neurons^6^,^8^,^9^. Interestingly, we find that activation of Or42b does not necessarily confer an attractive response. We show that two odorants that activate Or42b can have drastically different behavioral outputs for a second odorant tested shortly after, depending upon the temporal kinetics of the initial response. In this manner, the strong positive valence of this channel can be greatly impacted by prior odor exposures. It is known that different odorants are detected by the olfactory system by activating different combinations of receptors. However, we show the temporal kinetics of receptor activation may also play a role in how the animal perceives the odor landscape. These findings together have implications in understanding basic principles of odor coding in animals as well as for designing odor-based interventions to modify behavior.

## Results

### Or42b-mediated attractive valence depends upon prior odor exposure history

**Figure 1:**
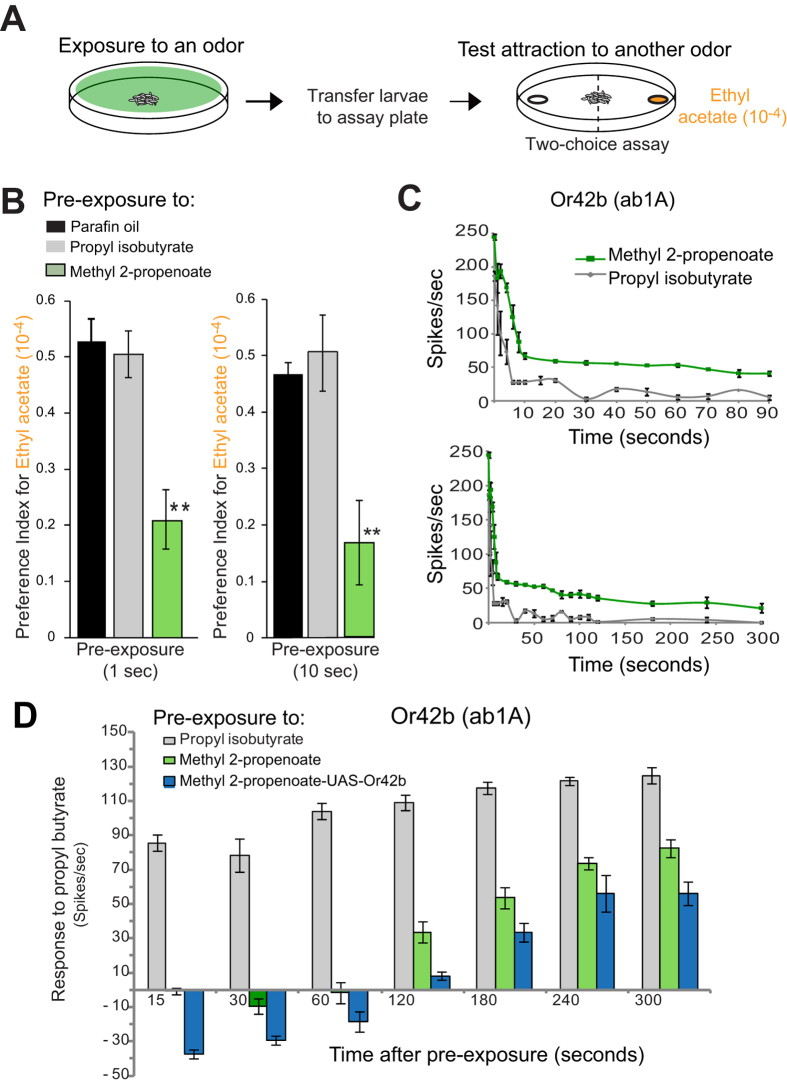
Prior odor exposure modifies subsequent odor valence. (**A**) Overview of pre-exposure screen to test odor that modifies subsequent olfactory behavior in a two-choice preference assay. (**B**) Preference index of *Drosophila* larvae to Ethyl acetate 10^−4^ after pre-exposure to the indicated odors for either 1-sec (left) or 10-sec (right) immediately prior to the test. N = 10 (~40 larvae/ trial), error bars = s.e.m. (**C**) Mean long-term response of ab1A to a 0.5- sec stimulus of indicated odor at t = 0. Each response curve is depicted in 2 separate graphs with different time windows, 90 sec and 300 sec. N = 3, error bars = s.e.m. (**D**) Mean changes in frequency of the ab1A to the indicated odor applied at indicated time points after pre-exposure to 0.5-sec odor stimulus indicated: grey = activator, green = prolonged-activator, blue = prolonged-activator response in *UAS-Or42b* expressing ab3A neurons (*ΔHalo; Or22a-G4,UAS-Or42b* flies). N = 5, error bars =  s.e.m.

While Or42b is a dedicated attraction channel we were surprised to find that a short 1-second exposure to one of its activators (methyl 2-propenoate) caused a dramatic reduction in subsequent attraction to another activator (ethyl acetate) (Fig. 1B). At this low concentration (10^−4^ dilution) ethyl acetate primarily activates Or42b and is attractive due to activation of this ORN^6^. Exposure to another equally strong activator of Or42b (propyl isobutyrate) did not reduce attraction, suggesting that the phenomenon was not simply related to adaptation of the Or42b ORN (Fig. 1B).

In order to examine this phenomenon at the neuronal level, we chose to examine Or42b responses in the analogous adult ab1A neuron since such analysis in the larval dome sensillum with 21 neurons is not technically feasible. Single unit electrophysiology indicated that exposure to a 0.5-second pulse of methyl 2-propenoate elicits a strong phasic response in the ab1A neuron, followed by a decrease to approximately half-maximal frequency in about 8–10 seconds, after which a relatively steady firing rate of ~60 spikes/sec above spontaneous activity remains for several minutes (Fig. 1C, green line). Responses to other equally strong activators return to baseline between 2–6 seconds after odor exposure (Fig. 1C, grey line)^10^,^11^,^12^. The prolonged response appears stronger than even activity evoked by a continuous odor pulse of 30-secs^10^ and appears similar to a class of pyrizine ligands identified for Or33a and Or59b^13^.

Surprisingly, the brief 0.5-second pre-exposure to methyl 2-propenoate subsequently renders the neuron unresponsive to changes in concentration of another activating odorant that it normally responds to. This decrease lasts the entire duration of the recording (300 seconds) (Fig. 1D, green bars). Masking is odor-specific since other equally strong activators do not have this effect (Fig. 1D, grey bars). Expressing *Or42b* in the “empty neuron” decoder system^14^ shows the same effect, indicating the effect is receptor-specific rather than neuron-specific (Fig. 1D, blue bars).

### Loss of attractive odor valence caused by prolonged tonic activation by previous odor exposure

**Figure 2:**
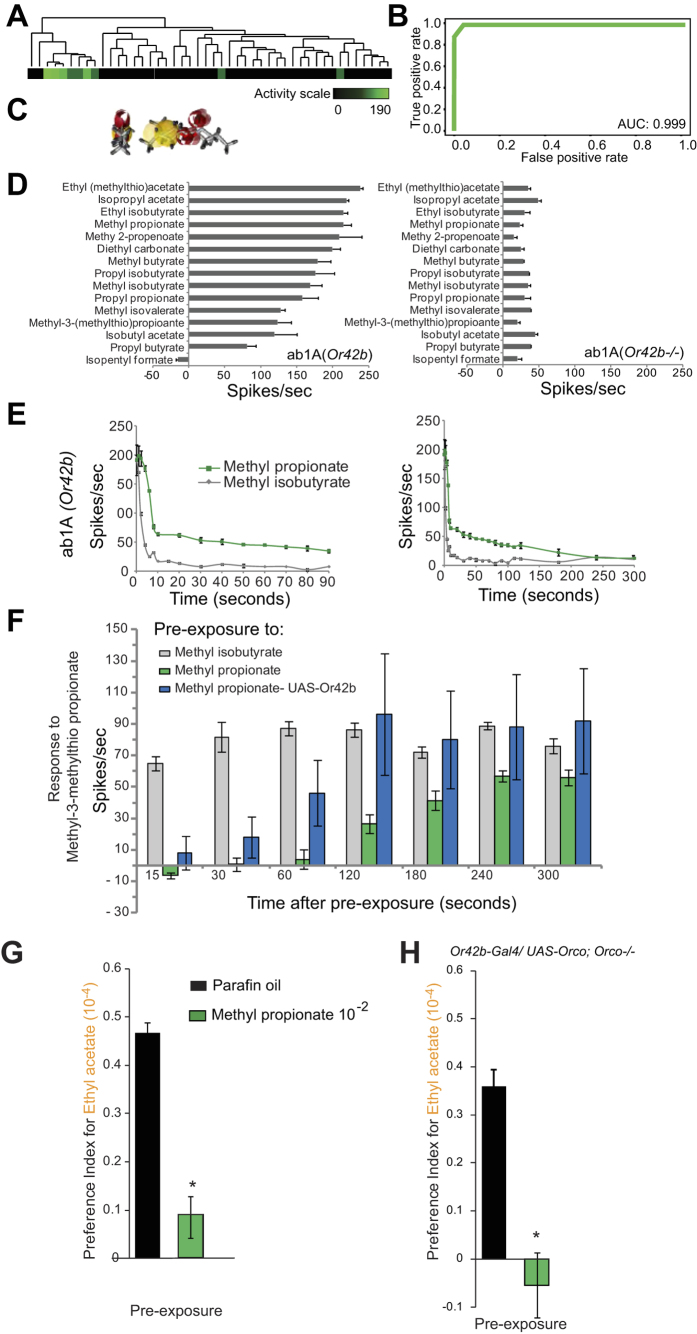
Modification of neuronal responses by pre-exposure to prolonged activators. (**A**) Cheminformatically determined ab1A neuron-optimized molecular descriptors can cluster known activating odorants from 46 odorant training set. (**B**) Computational validation of ligand predictive ability of the ORN-optimization approach. The mean true-positive value from 100 independent 4-fold cross validation runs of the support vector machine (SVM) approach is plotted as a receiver-operating-characteristic curve (ROC). (**C**) Pharmacophore of ab1A activators. (**D**) Identification of new ligands for ab1A (Or42b). Mean increase in response of ab1A to 0.5-sec stimulus of indicated cheminformatically predicted odorants in (left) wild-type and (right) Or42b^−/−^ flies (10^−2^ dilution). N = 3, error bars = s.e.m. (**E**) Mean long-term response of ab1A to a 0.5-sec stimulus of indicated odor at t = 0 over 90 sec and 300 sec. N = 3, error bars = s.e.m. (**F**) Mean changes in frequency of the ab1A to the indicated odor applied at indicated time points after pre-exposure to 0.5-sec odor stimulus indicated (grey = activator, green = prolonged-activator, blue = prolonged-activator response in UAS-Or42b expressing ab3A neurons in *ΔHalo; Or22a-G4,UAS-Or42b* flies). N = 5, error bars =  s.e.m. (**G**) Preference index of *wild-type* larvae, (**H**) *w;Or42b-GAL4/UAS-Orco; ΔOrco/ΔOrco* larvae to Ethyl acetate 10^−4^. Larvae were pre-exposed to the indicated odors for 10-sec immediately prior to the preference assays. N = 10 (~40 larvae/ trial), error bars = s.e.m. Students *t-*test. P-values =  *<0.05, **<0.001.

In order to test whether other prolonged activators also have similar behavioral masking effects, we identified several new activators of Or42b by utilizing a chemical informatics approach^15^. We used a training set of 47 odors whose activity had previously been tested in the *Or42b+* ab1A neurons^10^ and using a Sequential Forward Selection (SFS) approach identified an optimal subset of 13 molecular descriptors out of 3,224 (Dragon suite) that best describe the important structural features shared by activating odors (Supplementary Fig. 1). The optimized set of molecular descriptors could cluster activators together (Fig. 2A). These were then used to train a Support Vector Machine (SVM), an effective machine learning method, to perform predictions of ligands. We performed 100 independent 4-fold cross validations to computationally validate the predictive ability of the SVM, and a Receiver-Operating-Characteristic (ROC) curve was generated showing an Area Under Curve (AUC) value of 0.999 indicating high predictive success (Fig. 2B). This trained SVM was applied to computationally predict ligands from a collection of ~440,000 compounds resulting in a large ranked list of candidate agonists, some of which we hoped would demonstrate prolonged termination kinetics.

We selected 13 high-ranking predictions and tested them along with the two reported in Fig. 1 using electrophysiology on the ab1 sensillum. To our satisfaction, all of the predictions validated as *Or42b* dependent ligands, 12 agonists and 1 antagonist (Fig. 2D). From the new agonists we were able to identify an additional prolonged activator, methyl propionate, which showed a tonic response for nearly 220 seconds after a 0.5 sec stimulus (Fig. 2E). Consistent with our expectation, pre-exposure to this prolonged activator subsequently renders the neuron unresponsive to another activating odorant that it normally responds to. This was also validated using the empty neuron system (Fig. 2F). Odorants can have different rates of stimulus dynamics that can influence the duration of response^16^. We identified two additional odorant receptor neurons (Or59b and Or22a) are also strongly activated by methyl propionate. However these do not show prolonged termination kinetics for methyl propionate suggesting that the prolonged response to the odorant is receptor-specific (Supplementary Figure 2). This finding also suggests that simple physical explanations such as prolonged dynamics of odor release or residual chemical in the odor delivery tube, which could have caused continued activation is less likely.

Behavior trials with the newly identified prolonged activator, methyl propionate, clearly show that pre-exposure to it can cause a dramatic reduction in subsequent attraction to ethyl acetate as was seen with the first prolonged activator (Fig. 2G). The ability of an animal to respond behaviorally to changes in concentration of a ligand along an odor gradient or navigate along an odor plume in the environment depends on both sensitivity and rapidity of detecting incremental changes in concentration. Since both these properties are severely compromised upon exposure to prolonged activators, we see a long-term effect on odor valence even after the animal is removed from the vicinity of the odorant. Although we have taken care to use a covered behavior arena with minimal airflow, one caveat is that odorants are likely to be carried around as plumes by convection currents and animal movement. It is difficult to accurately ascertain or predict the concentration of these plumes that reach the dome sensillum from moment to moment for a direct comparison with the electrophysiology recordings.

In order to test whether the change in behavior is caused directly by prolonged-activation of Or42b, we performed a similar pre-exposure experiment on larvae that were genetically manipulated to have only the pair of *Or42b* expressing ORNs of the Or family class functional in the larvae. The expression of the obligate co-receptor *UAS-orco* in only the *Or42b* expressing neurons was done in an *orco* mutant background. Pre-exposure to the prolonged-activator but not paraffin oil solvent completely disrupted attraction towards ethyl acetate suggesting that ligand interaction with the Or42b receptor alone is sufficient to produce this masking effect (Fig. 2H, Supplementary Fig. 3).

### Prolonged activators are widespread across Or receptors

**Figure 3:**
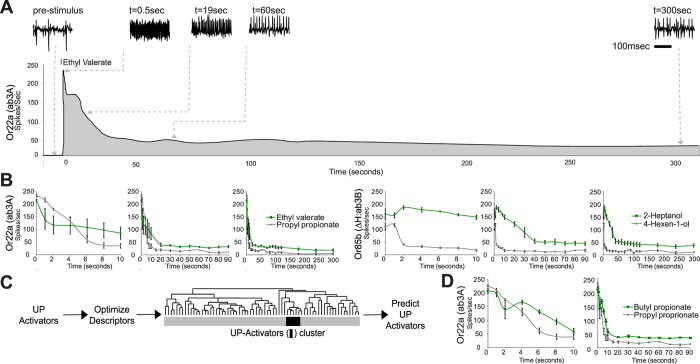
Prolonged activators can be identified with chemical informatics. (**A**) Long-term response of a single Or22a expressing ab3A neuron to a brief 0.5-sec stimulus of ethyl valerate (indicated as a red bar). Action potentials from 0.25-sec windows shown from indicated regions of the response. (**B**) Mean long-term response of indicated receptor expressing neuron to a 0.5-sec stimulus of indicated odor applied at t = 0 in 3 different time windows, 10 sec, 90 sec and 300 sec. For ease of spike counting ab3B (Or85b) recordings were performed in a ΔHalo (ΔH) mutant background^14^ where ab3A neuron is unresponsive. N = 3, error bars = s.e.m (**C**) Schematic representing the identification of prolonged activator-specific molecular descriptors that can cluster prolonged activators together in a tree containing all other Or22a activators. (**D**) Mean long-term response from an ab3A neuron expressing Or22a to a 0.5-sec stimulus of a predicted prolonged-activator depicted in 2 separate graphs with different time windows, 10 sec and 90 secs. N = 3, error bars = s.e.m.

This phenomenon is not restricted to the larvae. We also identified prolonged activators for additional Ors from the antenna by performing long-term recordings from several previously identified ligands for receptors expressed in the ab3 sensillum, *Or22a* and *Or85b*^11^,^15^ (Fig. 3A,B). Application of our chemical informatics approach on the Or22a receptors again identified descriptors that clustered prolonged activators and we successfully validated a new prolonged activator for Or22a (Fig. 3C,D). These results suggest that the optimized descriptors may be useful to distinguish odorants that cause prolonged responses even from amongst structurally similar odorants.

**Figure 4:**
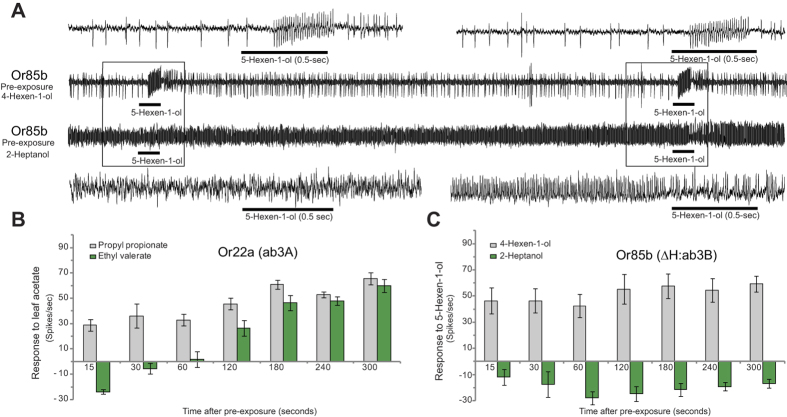
Prolonged activators affect other odorant receptors. (**A**) Representative electrophysiology traces of Or85b expressing ab3B neuron starting ~13 sec after a 0.5-sec pre-exposure to an activator (4-hexen-1-ol) or an prolonged-activator (2- heptanol) with repeated exposures to another activator (5-hexen-1-ol). Boxed area denotes 2-sec regions at 15 sec and 30 sec after pre-exposure where 0.5-sec of 5- hexen-1-ol (black bars) was applied, which are magnified above and below. Ab3B (Or85b) recordings performed in a ΔHalo (ΔH) mutant background^14^. (**B,C**) Mean increase in frequency of response of the indicated neuron to the indicated odor applied at indicated time points after pre-exposure to 0.5-sec odor stimulus (grey = activator, green = prolonged-activator). N = 5, error bars =  s.e.m. All odorants applied at 10^−2^ dilution.

As before, we find that a brief 0.5-second pre-exposure of these receptors to a prolonged activator renders the neuron unresponsive to changes in concentration of other activating odorants that the ORN normally responds to (Fig. 4A,B green bars). This masking effect again contrasts from pre-exposure to regular odorants where activation to comparable levels does not affect the ability of the ORN to detect subsequent repeated 0.5 second stimuli of an activating odorant (Fig. 4B, grey bars).

## Discussion

In *Drosophila* it is known that activation of a single glomerulus via a single ORN class is sufficient to drive behavior suggesting that each ORN “channel” is associated with a valence^8^,^17^,^18^. However, most odorants naturally activate more than one ORN “channel” and it is less clear how these channels interact to produce the observed behavioral outcome^5^,^6^,^9^,^11^,^12^.

Here we demonstrate a second level of sophistication in insect olfactory coding which allows an organism to evaluate odorants differentially based on the temporal activity of a single receptor. We find that exposure of the animal to some odorants can cause a prolonged activation of an attractive channel, which blocks subsequent activation of the same channel by more typical transiently activating compounds rendering these attractive odorants inactive. This effect is specific and does not occur with exposure to most activating odorants which do not have a prolonged tonic response. In this manner, the animal is able to use a single receptor, Or42b, to integrate information from two odor stimuli at the periphery and distinguish them based on differences in temporal kinetics.

This principle could be of immense value in blocking behavior of insect pests that transmit diseases to animals and cause agricultural damage. While antagonists have been found for Gr receptors^19^,^20^ it has been nearly impossible to identify antagonists for Or receptors that can block strong agonists, such as host cues. The *In silico* screens can thus provide a rational foundation for identification of prolonged activators of Ors that could act as novel insect repellents that can help in reducing insect-borne diseases.

## Materials and Methods

### Larval behavioral assay

The larval odor preference assays were performed as previously reported with some modifications^6^. 25uL of the odor solution or solvent was presented in inverted 0.2mL PCR tube caps. For pre-exposure experiments ~50 larvae per trial were placed on a 1% agarose base in a 90 mm disposable petri dish. 750 μl of odor solution was evenly spread on the lid and it was placed for indicated times (1 sec or 10 sec) on dish. Larvae were gently removed using a paint brush and placed on a separate petri dish and assayed for odor preference in the dark for 1 min 30 sec as described above.

### Fly lines used

Unless otherwise indicated, Canton-S backcrossed 5 times to w^1118^ was used as the wild-type control line (*wCS*) in this study. The mutant *Orco−/−* (previously called *Or83b*^1^)^7^ was obtained from Bloomington stock center, and the *Or42b-Gal4* and *UAS-Or42b* flies were a kind gift from Dr. John Carlson.

### Electrophysiology

Single unit electrophysiology recordings were performed as previously reported^14^ using 3–7 day old flies of the indicated genotypes. Diagnostic odorants were used to distinguish individual classes of ORNs in sensilla (ab1–ab7) and therefore unequivocally identify the target ORN for testing^10^,^12^. Odor stimulus flow = 12ml/second. The tested odorants were dissolved in paraffin oil at 1% dilution or their respective concentrations and 50uL of the odor solution was applied to cotton in a Pasteur pipette cartridge.

### Chemical informatics

Was performed essentially as before^15^ with the following changes. This ab1A(Or42b)-optimized descriptor set was used to train a Support Vector Machine (SVM) using regression and a radial basis function kernel available in the R package e1071, which integrates libsvm. A 5-fold cross-validation was performed dividing the training dataset randomly into 5 equal sized partitions. Four partitions were used to train the SVM and the remaining partition was tested for predictive ability. This process is repeated five times to cover the partitions and whole process is repeated 20 times to plot a receiver operating characteristics (ROC) curve. This trained SVM was utilized to predict new ligands from >440,000 small molecules obtained from the eMolecules database (www.emolecules.com) that has MW <325 and atoms: C, O, N, H, S.

## Additional Information

**How to cite this article**: Boyle, S. M. *et al.* Short-term memory trace mediated by termination kinetics of olfactory receptor. *Sci. Rep.* **6**, 19863; doi: 10.1038/srep19863 (2016).

## Supplementary Material

Supplementary Information
