# Supplementary material for: Short-term memory trace mediated by termination kinetics of olfactory receptor
Source: Sci Rep. 2016 Feb 1;6:19863. doi: 10.1038/srep19863 (PMC4735300; doi:10.1038/srep19863)
Supplement: Supplementary Information [file srep19863-s1.pdf]

TITLE: Short-term memory trace mediated by termination kinetics of olfactory receptor

AUTHORS: Sean Michael Boyle[1,*], Shane McInally[2,*], Sana Tharadra[2], Anandasankar Ray[1,2,3,4]

* these authors contributed equally

AFFILIATION:

[1] Genetics, Genomics and Bioinformatics Program.

[2] Department of Entomology.

[3] Center for Disease Vector Research.

[4] Institute of Integrative Genome Biology

University of California, Riverside, California, CA 92521

Optimized Descriptor List			
**Symbol**	**Description**	**Class**	**Dimensionality**
nRCOOR	number of esters (aliphatic)	Functional group counts	1
Mor10u	signal 10 / unweighted	3D-MoRSE descriptors	3
Mor04m	signal 04 / weighted by mass	3D-MoRSE descriptors	3
R1e+	R maximal autocorrelation of lag 1 / weighted by Sanderson electronegativity	GETAWAY descriptors	3
Mor27m	signal 27 / weighted by mass	3D-MoRSE descriptors	3
nHAcc	number of acceptor atoms for H-bonds (N,O,F)	Functional group counts	1
E1m	1st component accessibility directional WHIM index / weighted by mass	WHIM descriptors	3
GATS5m	Geary autocorrelation of lag 5 weighted by mass	2D autocorrelations	2
nROH	number of hydroxyl groups	Functional group counts	1
R5v	R autocorrelation of lag 5 / weighted by van der Waals volume	GETAWAY descriptors	3
Mor10p	signal 10 / weighted by polarizability	3D-MoRSE descriptors	3
C-006	CH2RX	Atom-centred fragments	1
Mor11e	signal 11 / weighted by Sanderson electronegativity	3D-MoRSE descriptors	3

Supplementary Figure 1. Optimized descriptor sets for ab1A (Or42b).
Optimized descriptor symbol, brief description, class, and dimensionality are listed. Descriptors are listed in ascending order of when they were selected into the optimized set.

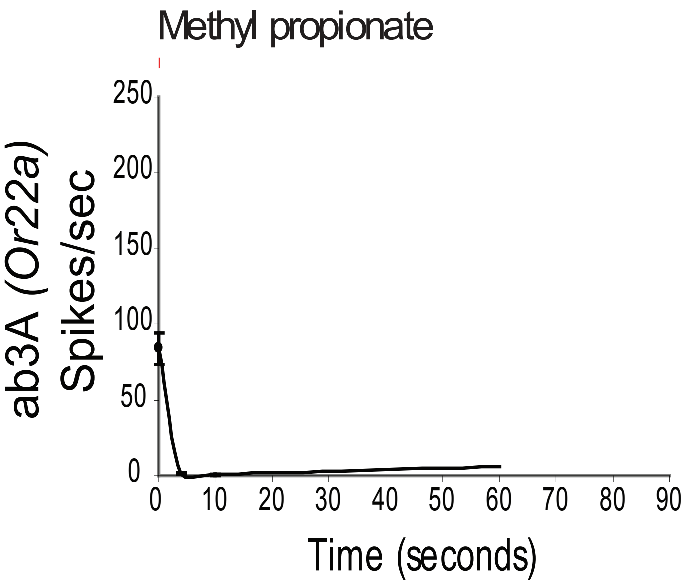

Supplementary Figure 2

Supplementary Figure 2. Not all receptors respond with prolonged tonic responses
Mean long-term response of indicated neurons to a 0.5- sec stimulus of indicated odorant applied at t=0 (10^{-2} dilution). Each response curve is depicted in 2 separate graphs with different time windows, 90 sec and 300 sec. N=3, error bars=s.e.m.!

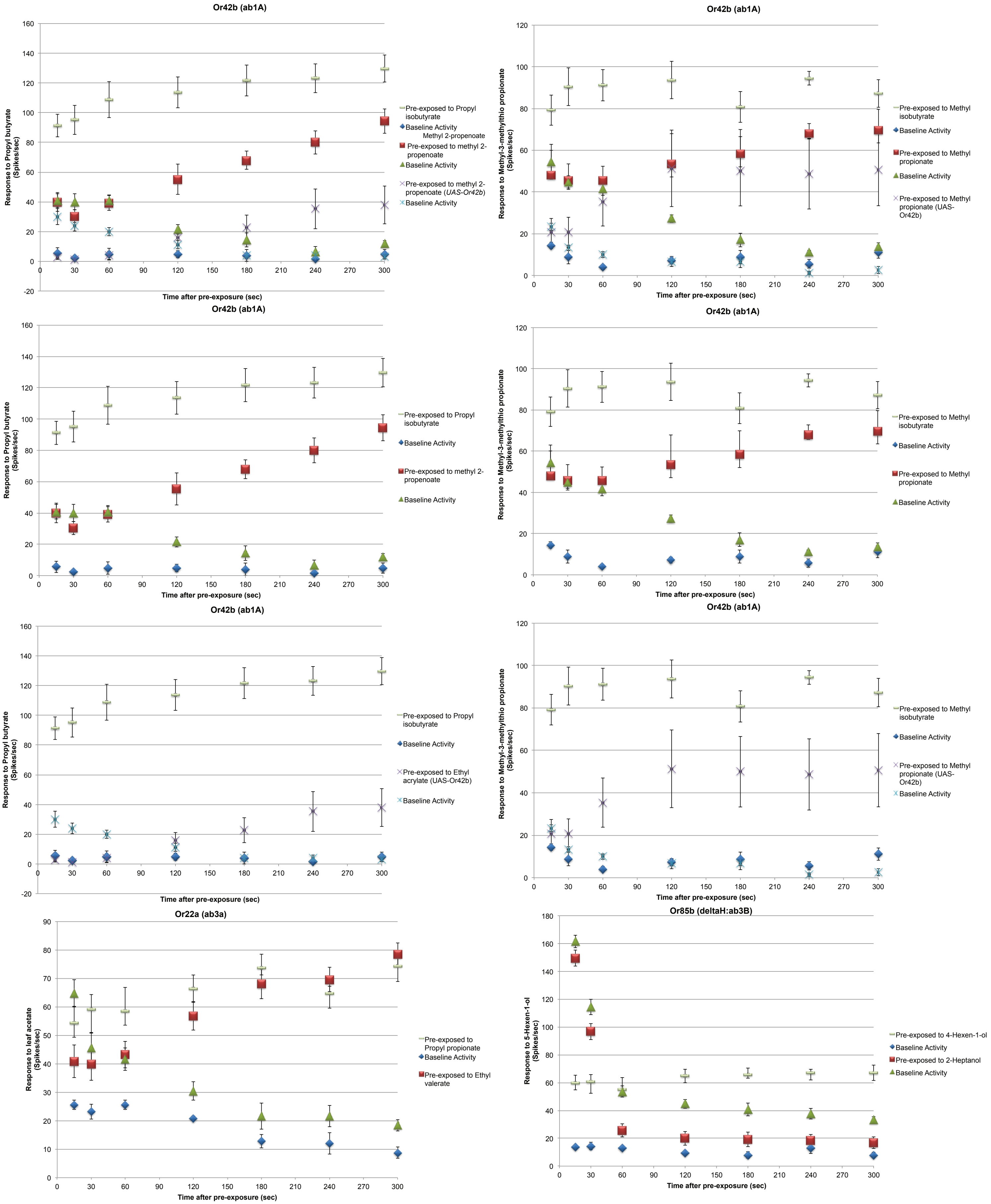

Supplementary Figure 3.
Mean activity of indicated neurons to a 0.5- sec stimulus of indicated odorant applied at t=0 (10^{-2} dilution). The mean baseline activity is indicated in these graphs, as opposed to main figures where the response during an odorant stimulus is reported as the increase in action potential frequency during the stimulus as compared to the 1-sec before the stimulus.
